# The bacterial community composition and its environmental drivers in the rivers around eutrophic Chaohu Lake, China

**DOI:** 10.1186/s12866-021-02252-9

**Published:** 2021-06-14

**Authors:** Keqiang Shao, Xin Yao, Zhaoshi Wu, Xingyu Jiang, Yang Hu, Xiangming Tang, Qiujin Xu, Guang Gao

**Affiliations:** 1 Taihu Laboratory for Lake Ecosystem Research, State Key Laboratory of Lake Science and Environment, Nanjing Institute of Geography and Limnology, Chinese Academy of Sciences, 210008 Nanjing, China; 2 School of Environment and Planning, Liaocheng University, 252000 Liaocheng, China; 3 Chinese Research Academy of Environmental Sciences, 100012 Beijing, China

**Keywords:** Lake Chaohu, River, Bacterial community composition, Functional profiles, COD_Mn_, NH_4_^+^

## Abstract

**Background:**

Bacterial community play a key role in environmental and ecological processes in river ecosystems. Rivers are used as receiving body for treated and untreated urban wastewaters that brings high loads of sewage and excrement bacteria. However, little is known about the bacterial community structure and functional files in the rivers around the eutrophic Chaohu Lake, the fifth largest freshwater lake in China, has been subjected to severe eutrophication and cyanobacterial blooms over the past few decades. Therefore, understanding the taxonomic and functional compositions of bacterial communities in the river will contribute to understanding aquatic microbial ecology. The main aims were to (1) examine the structure of bacterial communities and functional profiles in this system; (2) find the environmental factors of bacterial community variations.

**Results:**

We studied 88 sites at rivers in the Chaohu Lake basin, and determined bacterial communities using Illumina Miseq sequencing of the 16 S rRNA gene, and predicted functional profiles using PICRUSt2. A total of 3,390,497 bacterial 16 S rRNA gene sequences were obtained, representing 17 phyla, and 424 genera; The dominant phyla present in all samples were Bacteroidetes (1.4-82.50 %), followed by Proteobacteria (12.6–97.30 %), Actinobacteria (0.1–17.20 %). *Flavobacterium* was the most numerous genera, and accounted for 0.12–80.34 % of assigned 16 S reads, followed by *Acinetobacter* (0.33–49.28 %). Other dominant bacterial genera including *Massilia* (0.06–25.40 %), *Psychrobacter* (0-36.23 %), *Chryseobacterium* (0.01–22.86 %), *Brevundimonas* (0.01–12.82 %), *Pseudomonas* (0-59.73 %), *Duganella* (0.08–23.37 %), Unidentified *Micrococcaceae* (0-8.49 %). The functional profiles of the bacterial populations indicated an relation with many human diseases, including infectious diseases. Overall results, using the β diversity measures, coupled with heatmap and RDA showed that there were spatial variations in the bacterial community composition at river sites, and Chemical oxygen demand (COD_Mn_) and (NH_4_^+^ )were the dominant environmental drivers affecting the bacterial community variance.

**Conclusions:**

The high proportion of the opportunistic pathogens (*Acinetobacter*, *Massilia*, *Brevundimonas*) indicated that the discharge of sewage without adequate treatment into the rivers around Chaohu Lake. We propose that these bacteria could be more effective bioindicators for long-term sewage monitoring in eutrophic lakes.

**Supplementary Information:**

The online version contains supplementary material available at 10.1186/s12866-021-02252-9.

## Background

Rivers are the primary receiver of organic matter and nutrients from terrestrial ecosystems, and play a key role in biogeochemical cycles in aquatic ecosystems [1]. Although many studies emphasize the importance of river ecosystem services, most river worldwide have suffered serious deterioration, caused primarily by rapid industrialization and urbanization. Therefore, investigation of the overall ecological condition of the river ecosystems is of paramount importance.

In watersheds, lakes and their input rivers are highly linked in multiple ways, yet microbial diversity in river is less commonly studied than lake ecosystems [2]. As a crucial constituent of the river ecosystem, microbes are widely distributed in water column and are diverse in terms of numbers of species. They play a key role in the mineralization of organic matter, and biogeochemical processes [3, 4]. Although many studies have focused on bacterial community composition (BCC) in the water body of lakes [5–10], BCC in the input rivers around lakes has not been examined in as much detail. Futhermore, the bacterioplankton assemblage composition in lakes could be affected by the input of allochthonous bacteria [11]. In addition, rivers are also often used as receiving body for treated and untreated urban wastewaters [12], which brings high loads of sewage and excrement bacteria [13]. These bacterial genera usually include waterborne pathogens which are a danger to human health [14–16]. Nevertheless, the taxonomic and functional compositions of bacterial communities, and the influencing factors in the river around lakes have been largely ignored and the investigations are crying needed.

Our attention has been drawn to Chaohu Lake, the fifth largest freshwater lake in China, which is located in the downstream of the Yangtze River, which will serves many social, economic, and ecological purposes in the drainage basin [17, 18]. The western lake region receives major inflows, including the Nanfei and Shiwuli rivers (both have sewage outfalls), the Hangbu, and the Pai river. These western rivers account for almost 60 % of the total runoff volume contributed annually to the lake. The eastern lake region connects to the Yuxi river, an only outflowing river, which is the only channel connecting the eastern region to the Yangtze River. However, in the past decades, there has been increasing industrial and agricultural pollution and other strong human activities, causing serious deterioration in water quality of the lake, and increasing coverage and duration of cyanobacterial blooms [19]. Extensive research has been directed to the causes and mechanisms of eutrophication in Chaohu Lake [20]. However, no studies have characterized the bacterial community composition and functional profiles in the rivers around Chaohu Lake, nor has community structure been correlated with environmental factors.

In this study, excepted for investigating the composition of the bacterial community in the rivers around Chaohu Lake, we further studied which one is the environmental determinant of variations in the river bacterial communities. This work could decipher the spatial distribution patterns of BCC and its functional profiles in rivers around Chaohu Lake, and will be significant to understand the microbial ecology of the rivers and assessing ecological risk, as well as provide a scientific basis for the ameliorating pollution of the freshwater lake.

## Results

### Environmental characterization

The means, and maximum and minimum values, for the 11 environmental parameters measured at the 88 sampling sites in the rivers around the Chaohu Lake basin are summarized in Table 1. During the sampling period, the water temperature varies from 6 to 9 °C. Measurements related to trophic status typically varied greatly among sites. For example, TN concentration ranged from 0.71 mg L^− 1^ at C45 to 18.80 mg L^− 1^ at C25 (mean = 4.00 mg L^− 1^), TP concentration ranged from 0.03 mg L^− 1^ at C45 to 3.00 mg L^− 1^ at C25 (mean = 0.22 mg L^− 1^) and COD_Mn_ concentration ranged from 1.43 mg L^− 1^ at C81 to 16.71 mg L^− 1^ at C25 (mean = 5.02 mg L^− 1^). Concentrations of TN, TP and COD_Mn_ and other nutrients in the water column showed high levels of pollution in most of the rivers around Chaohu Lake. All water samples had a pH greater than 7, with the highest value of 9.66, indicating alkaline conditions in the study area (Table 1).

**Table 1:** Mean, and maximum and minimum values, for 12 physicochemical parameters for the 88 sampling sites in the rivers on 15 February 2018. Abbreviations are, *Temp* water temperature, *TN *total nitrogen, *DTN *dissolved total nitrogen, *NH*_4_^+^ ammonium, *NO*_2_^−^ nitrite, *NO*_3_^−^ nitrate, *TP* total phosphorus, *DTP *dissolved total phosphorus, *PO*_4_^+^ orthophosphate, *COD*_Mn_ Chemical Oxygen Demand, *BA *Bacterial abundance

Physicochemical parameters	mean	range
Temp (°C)	5.80	2.10-12.64
pH	8.56	7.60–9.66
TN (mg L^− 1^)	4.00	1.24–18.80
DTN (mg L^− 1^)	3.67	0.62–17.12
TP (mg L^− 1^)	0.22	0.03–1.47
DTP (mg L^− 1^)	0.15	0.01–2.57
NH_4_^+^ (mg L^− 1^)	1.82	0.01–18.7
PO_4_^+^ (mg L^− 1^)	0.04	0-0.66
NO_3_^−^ (mg L^− 1^)	1.75	0.05–7.98
NO_2_^−^ (mg L^− 1^)	0.05	0.01–0.50
COD_Mn_ (mg L^− 1^)	5.02	1.43–16.71
BA (cells mL^− 1^)	1.55 × 10^6^	1.52 × 10^4^ − 3.86 × 10^7^

### Patterns of bacterial abundance and diversity

**Fig. 1:**
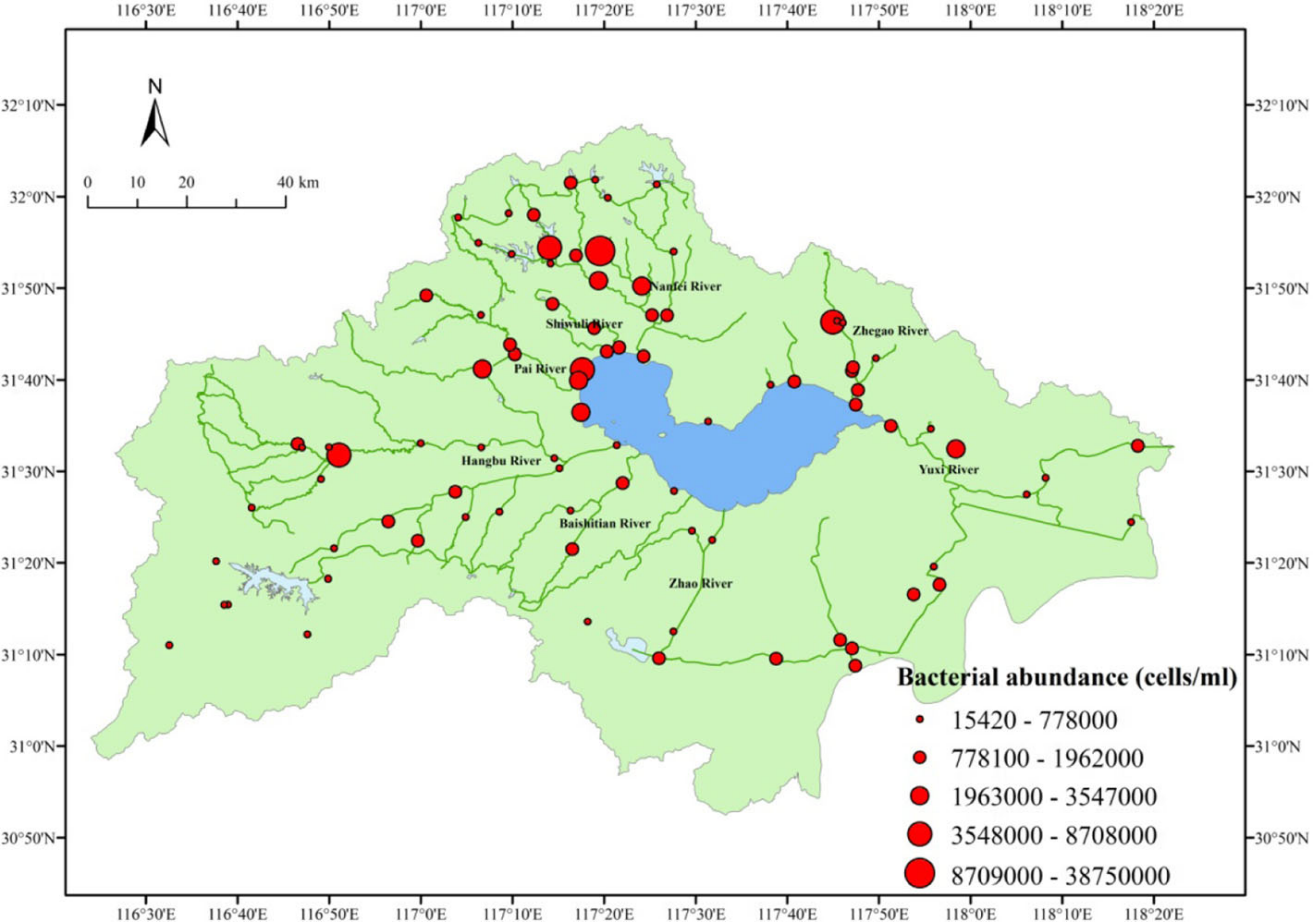
Spatial distribution of bacterial abundance at the 88 sites in the rivers around Chaohu Lake

Bacterial abundance varied greatly among the 88 sites in the rivers around the Chaohu Lake basin (Fig. 1). The bacterial abundance was lowest at site C64 with an abundance of only 1.52 × 10^4^ cells mL^− 1^, and highest in site C25 with an abundance of 3.86 × 10^7^ cells mL^− 1^; across all sites the mean abundance was 1.55 × 10^6^ cells mL^− 1^ (Table 1; Fig. 1).

**Fig. 2:**
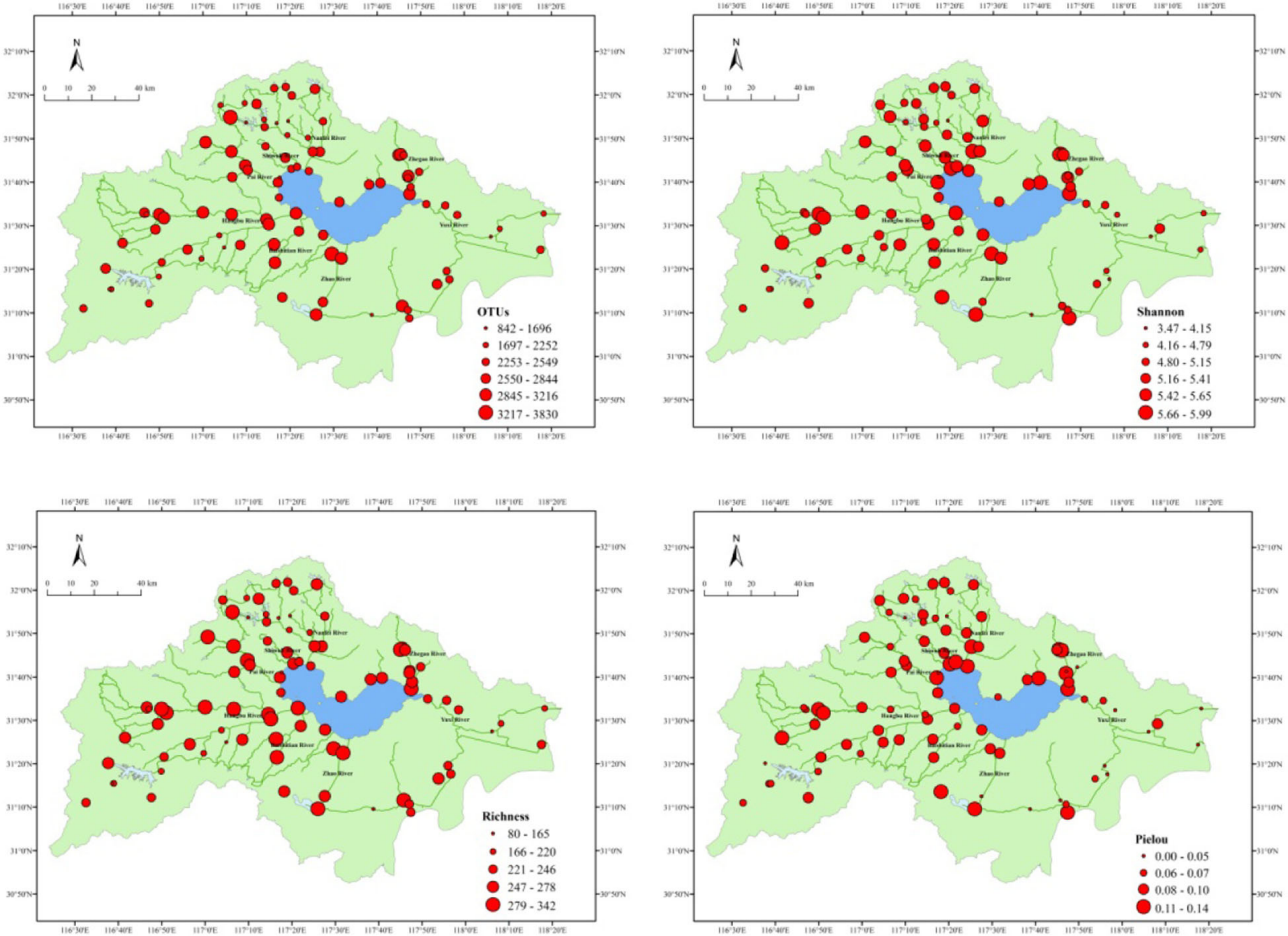
Spatial distribution of the OTUs and bacterial α-diversity, represented by Shannon, Richness and Pielou indices, of the 88 sites in the rivers around Chaohu Lake

Based on the identity level of 97 %, the 3,390,497 high quality sequence reads were classified into different OTUs after quality control. Among the 3,390,497 sequence reads, 125,901 OTUs were classified at the phylum level (Fig. 2). Rarefaction curves suggesting that the sequencing effort was sufficient to capture the community diversity (Fig. S1).

The bacterial α-diversity patterns, including the Richness, Shannon, and Pielou indexes were distinct between the 88 sites along the rivers (Fig. 2). The Shannon index ranged from 3.471 to 5.860, and was highest in sample C6. The Pielou index ranged from 0.02515 to 0.1378, and was highest in sample C6. The Richness index ranged from 79.74 to 308.30, with the highest value in sample C70.

**Fig. 3:**
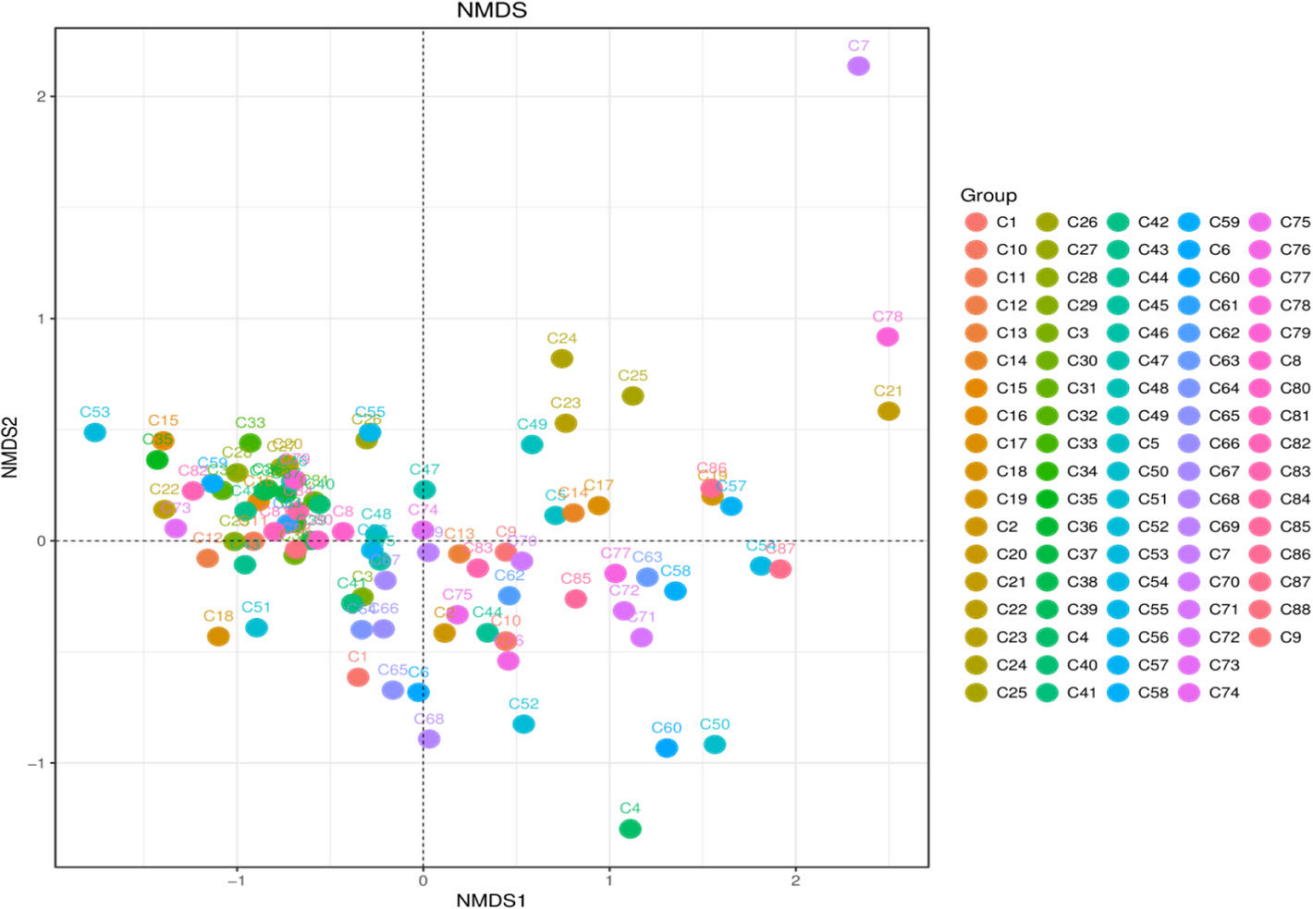
Non-metric multi-dimensional scaling (NMDS) plot based on the UniFrac weighted distance calculated from read numbers among the 88 sites around Chaohu Lake

We investigated the β-diversity patterns of the bacterial communities by employing non-metric multidimensional scaling (NMDS). Notably, these groups were scattered on the NMDS plot, suggesting lower similarity of bacterial community compositions among the 88 sites (Fig. 3). This result was also supported by the hierarchical cluster analysis, indicateing that the 88 samples were separated by site condition (Fig. S2).

### Phylogenetic composition of bacterial community

**Fig. 4:**
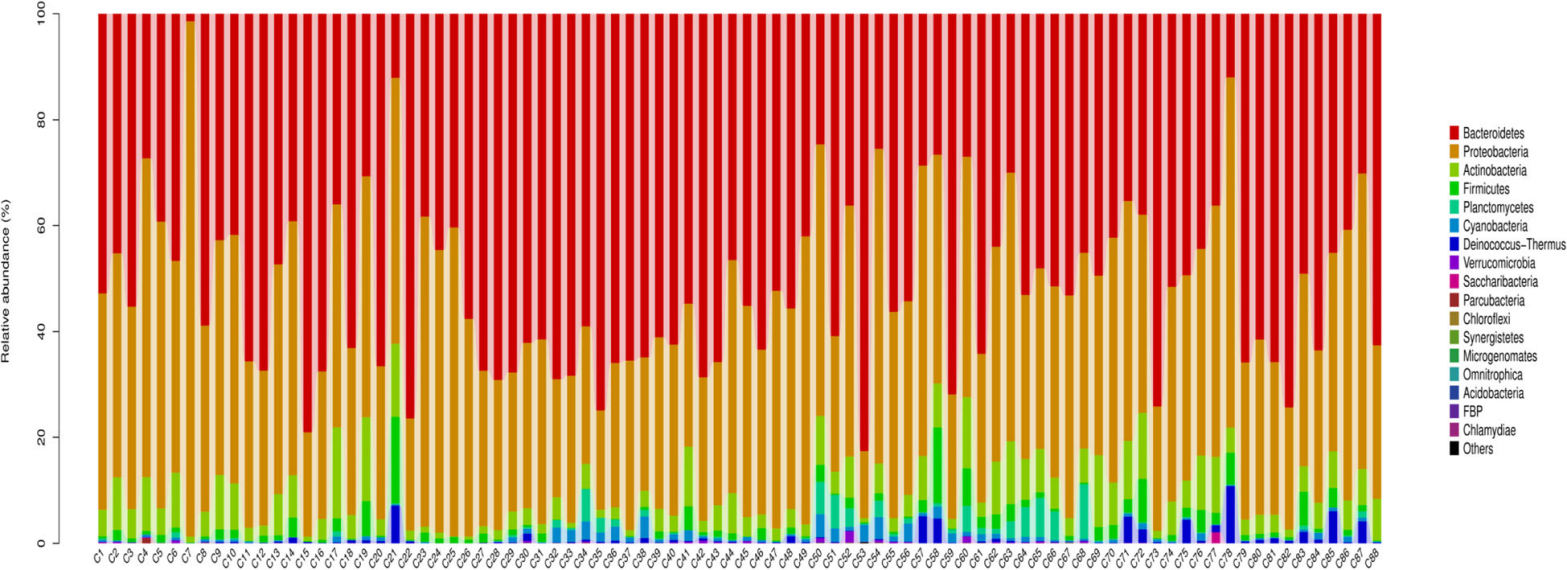
Profile of bacterial community composition, at the phylum level, in the 88 sites of the rivers around Chaohu Lake

**Fig. 5:**
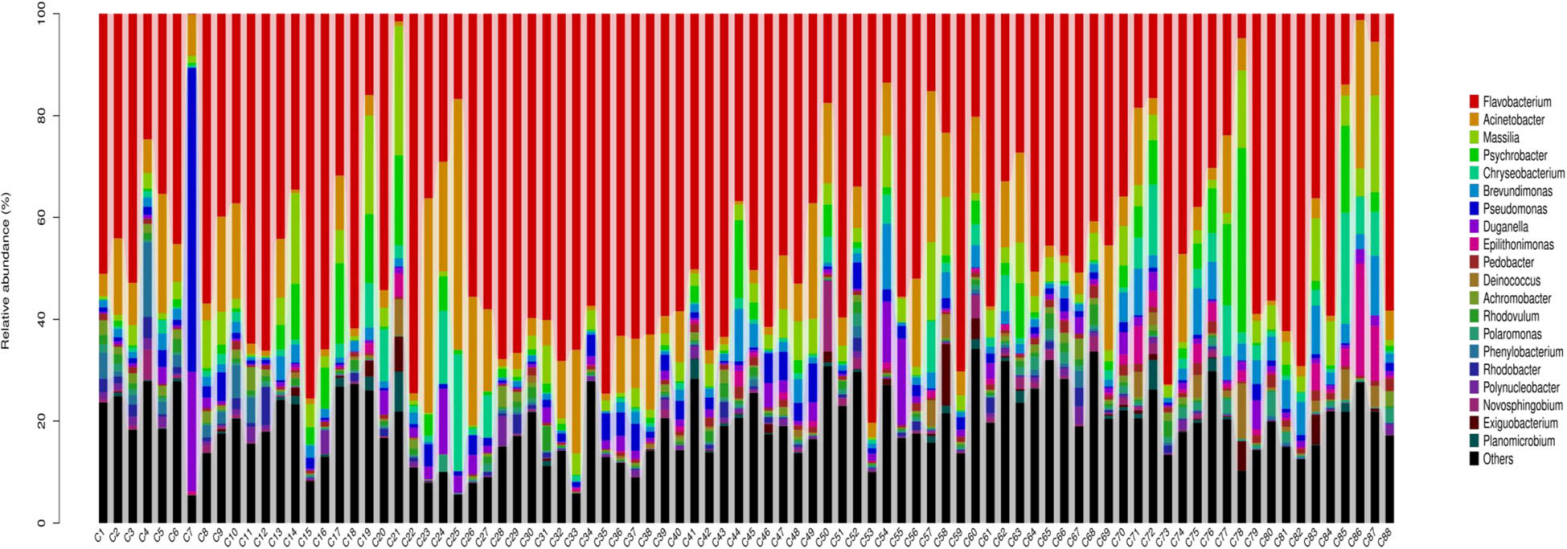
Profile of bacterial community composition, at the genus level, in the 88 sites of the rivers around Chaohu Lake

To visualize the bacterial community compositions in the rivers of the Chaohu Lake basin, the profiles of all taxa at all 88 sites were plotted, and are show in Fig. 4 (phylum level) and Fig. 5 (genus level). A total of 17 different phyla were observed, and the dominant bacterial phyla (those with ≥ 5 % relative abundance in any sample, average values of 88 sites) belonged to Bacteroidetes (51.6 %, average relative abundance), Proteobacteria (38.3 %), and Actinobacteria (5.5 %), which together accounted for 95.4 % of the bacterial sequences; the other phyla accounted for a low fraction of the average relative abundance (4.6 %). Among Proteobacteria, the most abundance class was Gammaproteobacteria, comprising 15.8 % of the total sequences, followed by Betaproteobacteria (13.5 %), and Alphaproteobacteria (9.0 %).

**Fig. 6:**
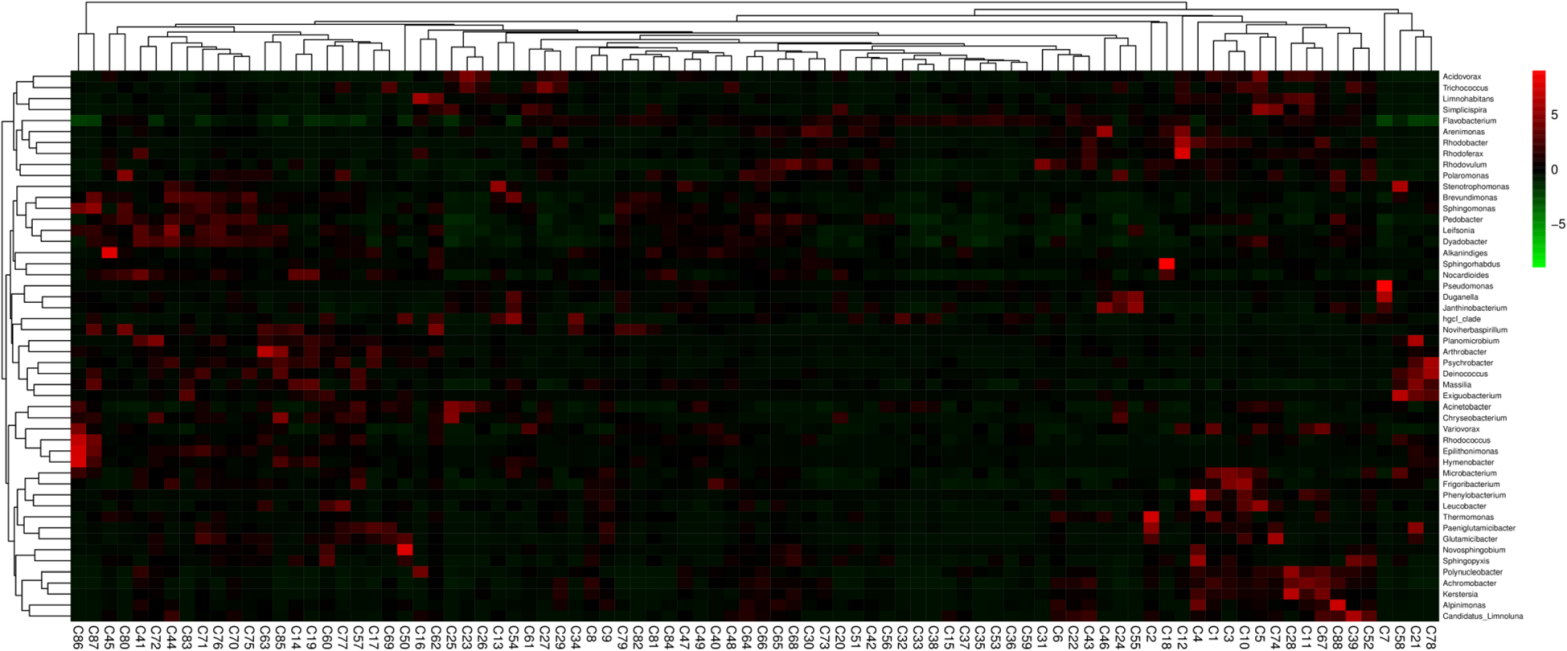
Heatmap profile showing 50 representative, predominant, 16 S rRNA gene-based sequences, classified at the genus level, using the Silva rRNA database

All reads were classified into 424 genus-level taxonomic groups, and 20 of them accounted for 80.89 % of all reads (Fig. 5). Among the 20 abundant genera, there were two genera of Bacteroidetes (48.3 %), followed by six Proteobacteria genera comprising α- (one genus), β- (two genera) and γ- subdivisions (three genera), and one genus of Actinobacteria. Deep taxonomic analyses showed that prominent genera (those with ≥ 1.4 % relative abundance in any sample, average values of 88 sites) consisted of *Flavobacterium* (45.6 %, average relative abundance), *Acinetobacter* (8.8 %), *Massilia* (4.6 %), *Psychrobacter* (3.2 %), *Chryseobacterium* (2.7 %), *Brevundimonas* (2.6 %), *Pseudomonas* (2.3 %), *Duganella* (2.1 %), and unidentified *Micrococcaceae* (1.4 %), which together accounted for 73.3 % of the bacterial sequences; other genera accounted for only 26.7 %. Heatmaps of the 50 most abundant bacterial genera based on the relative abundances indicated that more differences of bacterial community composition were observed between each site at the genus level (Fig. 6).

### Functional profiles of the bacterial community

**Fig. 7:**
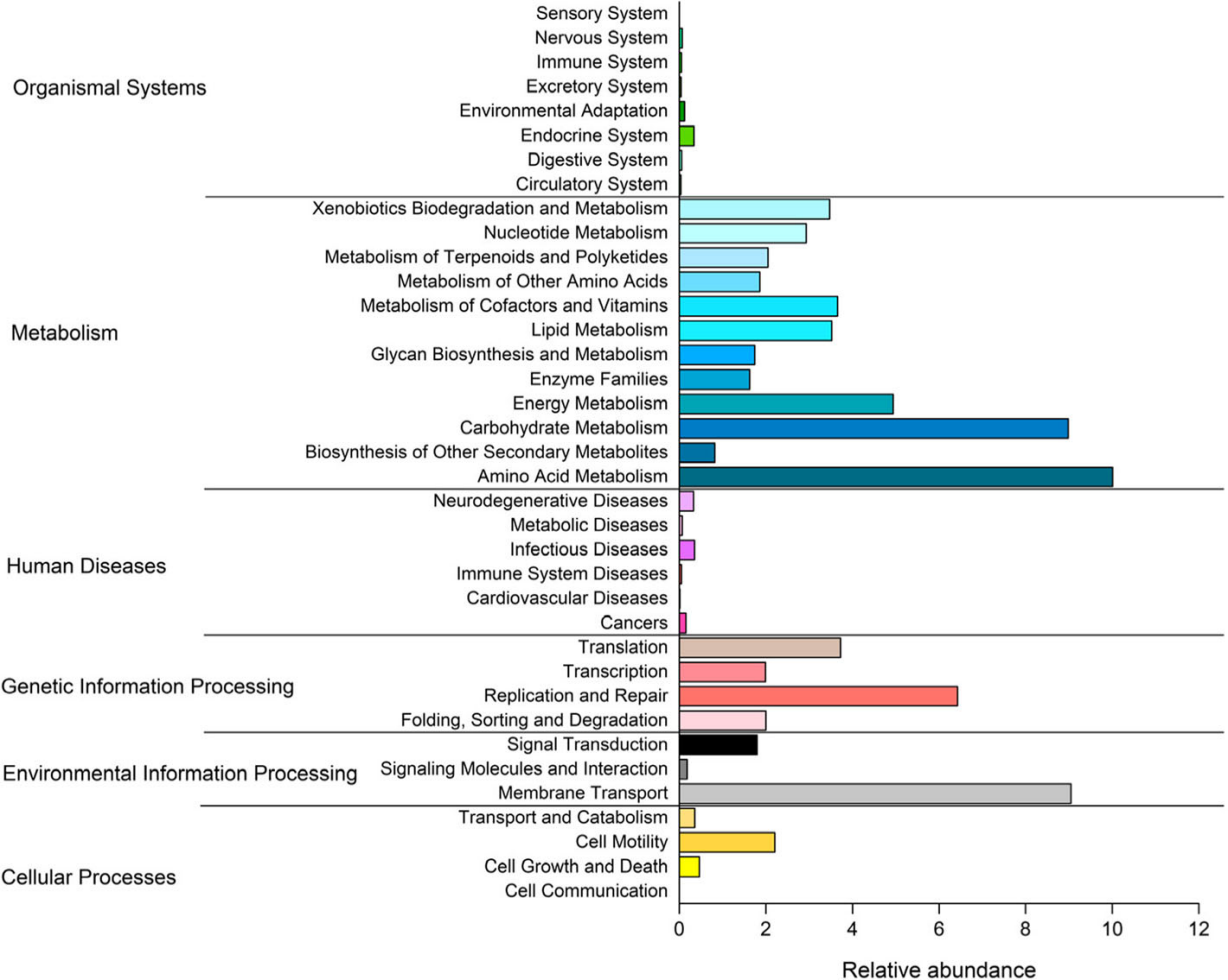
The relative abundance of predicted functions of bacterial communities in the rivers around Chaohu Lake, calculated using PICRUSt2 grouped into level-2 functional categories

The results of PICRUSt program based on the KEGG classification showed that the predominant predicted bacterial functions were those related to, metabolism, genetic information processing, environmental information processing, human diseases and organismal systems (Fig. 7). Due to the importance of water quality for human health, we further targeted bacterial functional classes related to human diseases. We found that predicated functions were related to six kinds of diseases, cardiovascular, infectious, immune, metabolic, neurodegenerative, and cancer (Fig. 7). Involvement with infectious diseases was the most dominant (Fig. 7).

### Linking bacterial community to environmental parameters

**Fig. 8:**
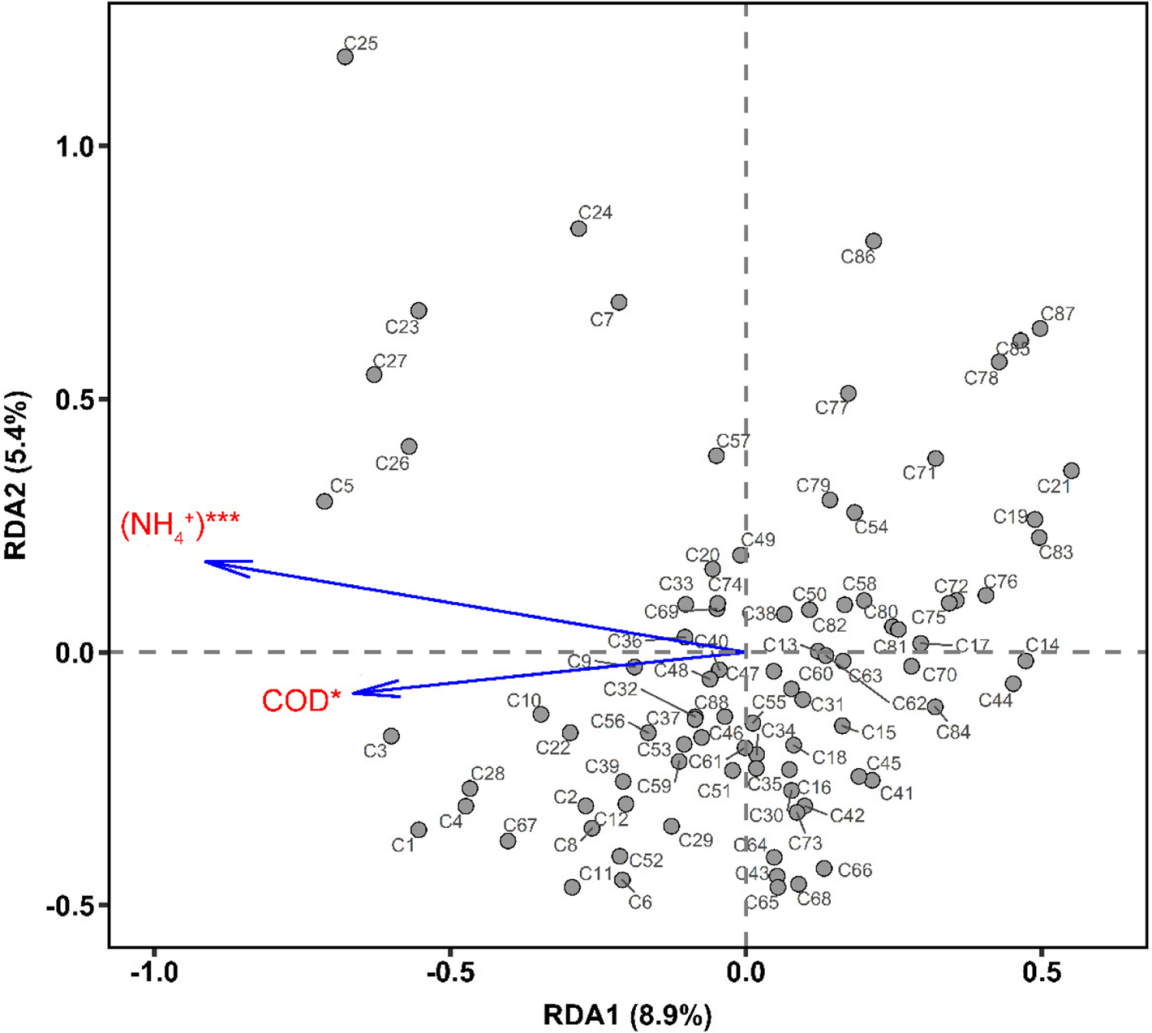
Redundancy analysis ordination plots show the relationship between two key environmental variables (COD_Mn_ and NH_4_^+^) and bacterial community structure, as reviewed by assessing 16 S rRNA gene OTUs. The nominal variable ‘‘sampling site’’ is represented as centroids. Environmental variables that are significant are shown by * (*P* < 0.05), and *** if very significant (*P* < 0.01)

To assess the correlation of community structure with environmental parameters, we performed a redundancy analysis (RDA) biplot of the BCC of 88 sites and 11 physicochemical parameters (temp, pH, TN, DTN, NH_4_^+^, NO_2_^−^, NO_3_^−^N, TP, DTP, PO_4_^+^, and COD_Mn_) (Fig. 8). The plot demonstrated that COD_Mn_ and NH_4_^+^ played a significant role in the spatial changes of bacterial communities of the rivers (Monte Carlo test *P* < 0.05). The first RDA dimension explained 8.9 % of the variation of bacterial communities, and the second explained 5.4 %.

## Discussion

### Dominant taxa groups in the BCC in the rivers

Bacteroidetes, Proteobacteria and Actinobacteria were the most abundant phyla in the rivers around the Chaohu Lake, China. The sum of the average relative abundances of these three phylum exceeded 95 % of the total community. Among them, Bacteroidetes occupied 51.6 %. These bacteria play key role in the degradation of protein, cellulose, pectin and chitin, which are typical components of the high molecular mass fraction of dissolved organic matter [7, 21, 22]. The high abundance of Bacteroidetes in the rivers may be related to the organic matter contamination and algal blooms from the study area [18, 23]. Previous study in the Tama River also showed that the soaring numbers of Bacteroidetes may be related to cyanobacterial blooms [7] and river pollution levels [24]. In terms of genera, *Flavobacterium* was the most dominant one (45.6 % among bacteria) in this group (Fig. 2), which has been related to harmful algal blooms because of its algicidal activity and antagonism to other bacteria [25]. Previous study also indicated that *Flavobacterium* was often found in high abundance in eutrophic and hypertrophic urban rivers [26].

Proteobacteria was another predominant phylum, with Gammaproteobacteria, Betaproteobacteria, and Alphaproteobacteria predominating in all samples. Proteobacteria is the largest phylum of bacteria, and is involved in a variety of biogeochemical processes in aquatic ecosystems [27, 28]. Deep taxonomic analyses revealed that the genera *Acinetobacter*, *Massilia*, and *Brevundimonas* were the main components of the Gammaproteobacteria, Betaproteobacteria, and Alphaproteobacteria respectively, accounting for 8.8 %,4.6 and 2.6 % of all reads. Examination of the scientific literature showed they are the opportunistic pathogens and infectious [14, 18, 29]. In this study, the high abundance of *Acinetobacter*, *Massilia*, *Brevundimonas* may be related to failure in sewage treatment processes that may be reflected in the high concentration of TN、TP and COD_Mn_ of the river water environment. Previous studies also showed that the occurrence of pathogenic bacteria in river following failure in sewage treatment processes may increase near large urban populations [30, 31].

Actinobacteria was the third dominant phylum in our study. Actinobacteria are widely distributed and are the most abundant phylum in freshwater ecosystems [32, 33]. Deep taxonomic analyses showed that the main component of the Actinobacteria was the genus “unclassified *Micrococcaceae”*, which accounted for 88.1 % of the relative abundance. In total, 1.4 % of them were “unclassified *Micrococcaceae* within the family *Micrococcaceae”*. This finding is of special local note, and may reflect a particular local food. In the processing of dry-fermented sausages, *Micrococcaceae* are the crucial microorganisms used as starter cultures, and are used in the preservation of meat products that avoid rancidness and develop the typical red colour owing to catalase and nitrate reductase [34]. The Chaohu Lake lies in the Anhui Province of China, where preserved bacon is local speciality food [35].

### Functional profiles of bacteria in the rivers

Understanding the functional profiles of bacterial communities is of great importance because it may shed light on ecosystem processes and community assembly mechanisms [1]. In our present study, functional profiles using PICRUSt revealed that the bacteria in the rivers of the Chaohu lake were involved in many diverse pathways (Fig. 4), most of which were related to metabolism systems, genetic information processing and environmental information processing. In addition, a considerable group of bacteria were involved in human diseases, including infectious, neurogenerative, metabolic, immune system, cardiovascular diseases and cancer. Among these, the most dominant was involvement in infectious diseases. This finding is consistent with previous finding in the Apies River, in South Africa [36].

The occurrence, and increase of bacteria associated with human diseases in river water may be related to the release of effluent from wastewater treatment plants, ineffective septic tank systems, and storm water runoff [37]. Although human pathogenic bacteria often occur at low levels in the environment [38], the pollutants that enter these systems may settle into the bottom sediments, and pathogenic bacteria will gradually increase in numbers, and finally increase the risk of infections to humans and animals [36]. Therefore, effective governance of untreated sewage in an urban river is of paramount importance.

### Response of the community structure of river bacteria to COD_Mn_ and NH_4_^+^

We found that there were significant differences in bacterial community composition among the 88 sites in the rivers (Figs. 3 and 6). This agrees with other findings that there is variation largely in the taxonomic composition and spatial distribution of freshwater bacterial community among different rivers [39–43]. Our RDA results revealed that COD_Mn_ and NH_4_^+^ were the most significant determining factors related to the variances in the river bacterial community around the Chaohu Lake. These may relate strongly to the significant variations of COD_Mn_ and NH_4_^+^ concentrations at different rivers. A correlation between COD_Mn_ and NH_4_^+^ and bacterial community structure has been reported previously. In a 2008 study, Wei et al. found that bacterial community structures in Chaohu Lake were influenced significantly by the influent COD_Mn_ [9]. Previous studies in China have also revealed that COD_Mn_ was significantly related to the bacterial composition diversity in an urban river [44], and COD_Mn_ and NH_4_^+^ were significant structuring factors for bacterial community compositions in an urban lake [45].

## Conclusions

In summary, using Illumina miseq sequencing, we explored bacterial community diversity, composition and functional profiles of 88 sites in the rivers around Chaohu Lake, China. The results showed that Bacteroidetes, Proteobacteria and Actinobacteria were the dominant phyla, and dominant genera included *Flavobacterium*, *Acinetobacter*, *Massilia*, *Psychrobacter*, *Chryseobacterium*, *Brevundimonas*, *Pseudomonas*, *Duganella*, and *Unidentified Micrococcaceae*. The functional profiles of the bacterial populations revealed an association with many human diseases, including infectious diseases. We also found site differences in the bacterial community structure in river water, COD_Mn_ and NH_4_^+^ were the main drivers regulating these variations. Our results indicated that the discharge of sewage without adequate treatment into the rivers around the Chaohu Lake owing to widespread occurrence of pathogenic bacteria (*Acinetobacter*, *Massilia*, *Brevundimonas*), and these bacteria could be more effective bioindicators for long-term sewage monitoring in eutrophic lakes. Therefore, the capacity of sewage treatment needs to be substantially strengthened around the Chaohu watershed to protect Chaohu Lake from further contamination.

## Methods

### Study area and sampling

**Fig. 9:**
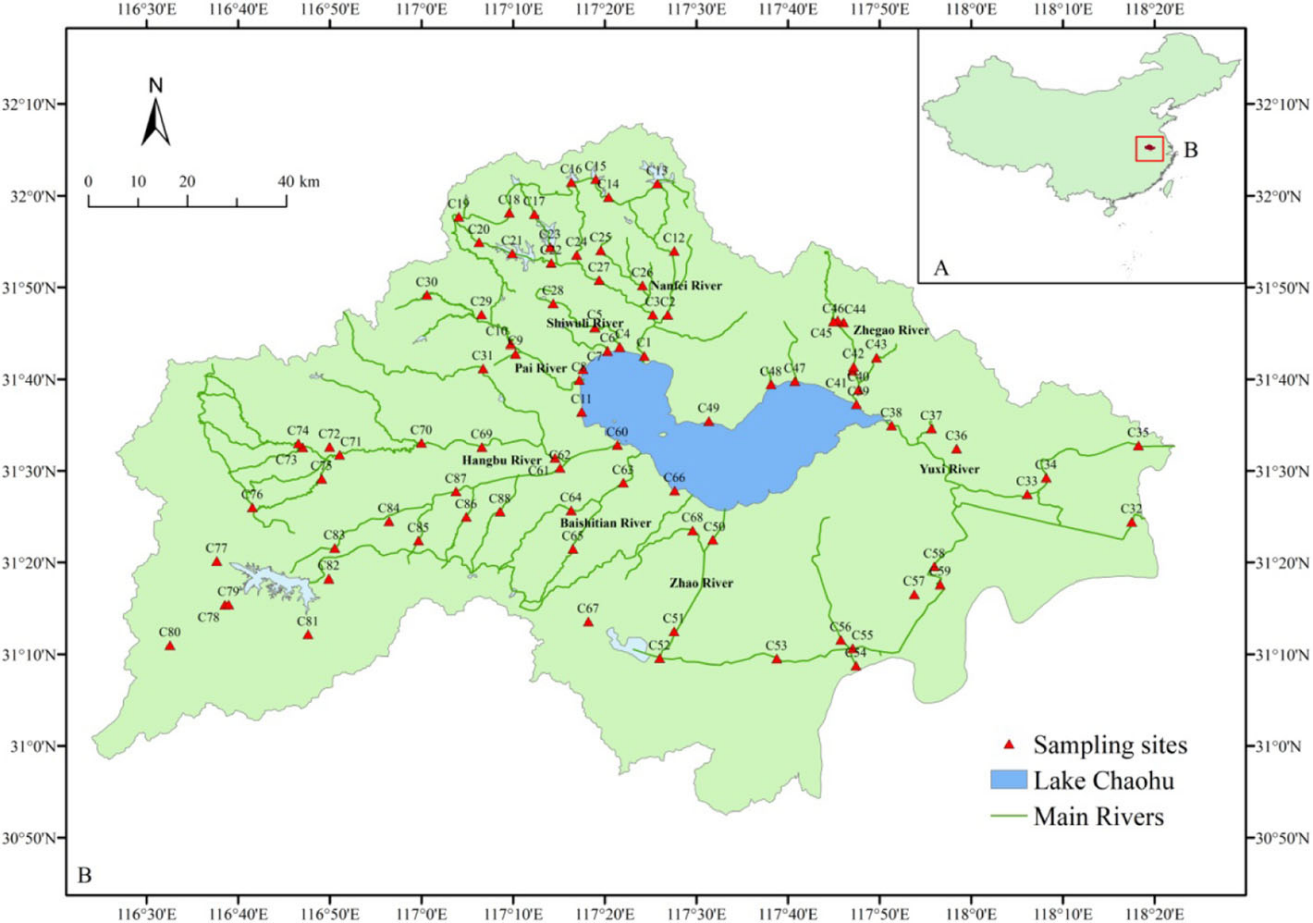
Map showing the location of the 88 sites in the rivers around Chaohu Lake

Chaohu Lake (31°25′- 31°43′N, 117°16′- 117°51′E), is located in the center of Anhui Province, China, and in the downstream of the Yangtze River. The lake has a surface area of 760 km^2^ and can be divided into two regions, from the Zhongmiao Temple to Qitouzui Cape (Fig. 9), the western region is eutrophic to hypertrophic, and the eastern region is mesotrophic. The western region receives major inflows, including the Nanfei and Shiwuli rivers (both have sewage outfalls), the Hangbu, and the Pai river. These western rivers account for almost 60 % of the total runoff volume contributed annually to the lake. The eastern region connectes to the Yuxi river, which is the only channel connecting the eastern region to Yangtze River, permitting water exchange [18, 46].

On 15 February 2018 we carried out our field work at 88 sites of the rivers around the Lake Chaohu basin (Fig. 9). At each sites, surface water (top 50 cm) was collected with a 5 L Schindler sampler. For 16 S rRNA gene analysis, a subsample of water (500 mL) was pre-filtered in situ with polycarbonate membrane (0.2 μm pore-size and 47 mm diameter, Millipore) using a hand-driven vacuum pump. These filters were frozen at -80 ^o^C until DNA extraction was performed. For enumeration of bacterial abundance, an additional subsample (46 mL) was transferred into an autoclaved tube containing 4 mL of prefiltered (pore size, 0.2 μm) glutaraldehyde (final concentration 2 % [v/v]). These samples were then stored in a refrigerator at 4 ^o^C, until slides were prepared for enumeration of bacterial abundance. The remaining water samples were transported to the laboratory in dark cooling boxes, and processed 3–5 h after sampling within 4 h for immediate chemical analysis.

### Measurement of environmental parameters and bacterial abundance

Water temperature (Temp) and pH were measured in situ using a multi-parameter water quality sonde (YSI 6600V2, Yellow Springs Instruments, USA). Chemical analyses of water samples for nine parameters (total nitrogen (TN), dissolved total nitrogen (DTN), ammonium (NH_4_^+^), nitrate (NO_3_^−^), nitrite (NO_2_^−^), total phosphorus (TP), dissolved total phosphorus (DTP), orthophosphate (PO_4_^−^), and chemical oxygen demand (COD_Mn_) were conducted in the laboratory according to standard methods. The abundance of bacteria in the water samples was determined by the 4’,6’-diamidino-2-phenylindole (DAPI)-combined epifluorescence direct counting method [47].

### DNA extraction, PCR amplification and Illumina Miseq sequencing

The total DNA was extracted using proteinase K, sodium dodecy1 sulfate, and cetyltrimethyl ammonium bromide, follow by phenol-chloroform extraction and isopropanol precipitation [48]. Crude DNA extracts were then purified by the E.Z.N.A® cycle-Pure kit (Omega Bio-Tek).

The V4-V5 regions of the 16 S rRNA genes were amplified using the primers 515 F (GTGCCAGCMGCCGCGGTAA) and 907R (CCGTCAATTCMTTTRAGTTT). Polymerase chain reaction (PCR) amplification was performed in a 25 µL reaction mixture containing 5 µL of 5 × PCR buffer, 1 µL of each primer (10 µmol L^− 1^ each), 20 ng µL^− 1^ DNA template, and 0.25 µL of *Taq* polymerase (5 U µL^− 1^; Fermentas). The PCR cycling was performed in a thermocycler (Applied Biosystems Veriti Thermal Cycler) under the following conditions: 98 °C for 2 min, 28 cycles each at 98 °C for 15 s, 55 °C for 30 s and 72 °C for 30 s; with final extension at 72 °C for 5 min. Sequencing service was performed by an Illumina Miseq platform at Personal Biotechnology Co., Ltd (Shanghai, China).

### Sequencing data processing

Sequence reads were processed by the Quantitative Insights Into Microbial Ecology (QIIME) v. 1.9.1 pipeline [49]. After quality controlling, bacterial phylotypes were assigned to operational taxonomic units (OTUs, 97 % cutoff) using the Uclust algorithm [50]. The longest sequence in each cluster was chosen as the representative sequence, which were annotated by the Silva rRNA database project (SILVA VERSION SSU11; http://www.arb-silva.de/).

### Predictive metagenome analysis

The metagenome functional content was predicted using the Phylogenetic Investigation of Communities by Reconstruction of Unobserved States (PICRUSt) software package (version 2.0.0, https://github.com/picrust/picrust2/wiki) [51]. PICRUSt2-compatible OTU tables were made using the closed-reference OTU picking protocol in QIIME against the RDP database. The nearest sequenced taxon index was used as a measure to represent the novelty of bacteria within an OTU table, in respect of previous sequenced genomes. The obtained OTU table was normalized as the true abundance, and applied predict_metagemones.py with default settings to obtain the predicted metagenomics table with Kyoto Encyclopedia of Genes.

### Statistical analysis

Bacterial alpha-diversity was processed using the QIIME pipeline. Community differences between sites were visualized by non-metric multidimensional scaling (NMDS) that was performed using the R statistical program [52]. Heat maps of the most abundant bacterial genera were analyzed using a “pheatmap” package in the R environment. Correlations between environmental variables and bacterial communities were measured with ordination methods using the vegan package in the R environment.

## Supplementary Information

**Additional file 1: Figure S1. **Rarefaction curves of the number of operational taxonomic units (OTUs) at 97% similarity boxplot for each of 88 samples. **Figure S2.** UPGMA result based on the unweighted Unifrac metric. The hierarchical clustering structure helps to determine the similarity of the bacterial communities between different samples.

## Data Availability

All data generated or analyzed during this study are included in this published article and its supplementary information files. The raw data are available from the corresponding author on reasonable request. Sequence data of this project have been deposited in the Sequence Read Archive (SRA) of the National Center for Biotechnology Information (NCBI), with accession number SRP189003 (Persistent web link to datasets: https://www.ncbi.nlm.nih.gov/sra/?term=SRP189003).

## Abbreviations

BCC: Bacterial community composition
COD_Mn_: Chemical oxygen demand
DTN: Dissolved total nitrogen
DTP: Dissolved total phosphorus
KEGG: Kyoto Encyclopedia of Genes
NCBI: National Center for Biotechnology Information
NH_4_^+^: Ammonium
NMDS: Non-metric multidimensional scaling analysis
NO_3_^−^: Nitrate
NO_2_^−^: Nitrite
OTU: Operational Taxonomic Units
PCR: Polymerase chain reaction
PERMANOVA: Permutational Multivariate Analysis of Variance
PICRUSt: Phylogenetic Investigation of Communities by Reconstruction of Unobserved States
PO_4_^+^: Orthophosphate
QIIME: Quantitative Insights Into Microbial Ecology
TN: Total nitrogen
TP: Total phosphorus
