# Supplementary material for: The bacterial community composition and its environmental drivers in the rivers around eutrophic Chaohu Lake, China
Source: BMC Microbiol. 2021 Jun 14;21:179. doi: 10.1186/s12866-021-02252-9 (PMC8201733; doi:10.1186/s12866-021-02252-9)

**Figure legends**

**Figure. S1** Rarefaction curves of the number of operational taxonomic units (OTUs) at 97% similarity boxplot for each of 88 samples.

**Figure. S2** UPGMA result based on the unweighted Unifrac metric. The hierarchical clustering structure helps to determine the similarity of the bacterial communities between different samples.

**Figure S1.**

**
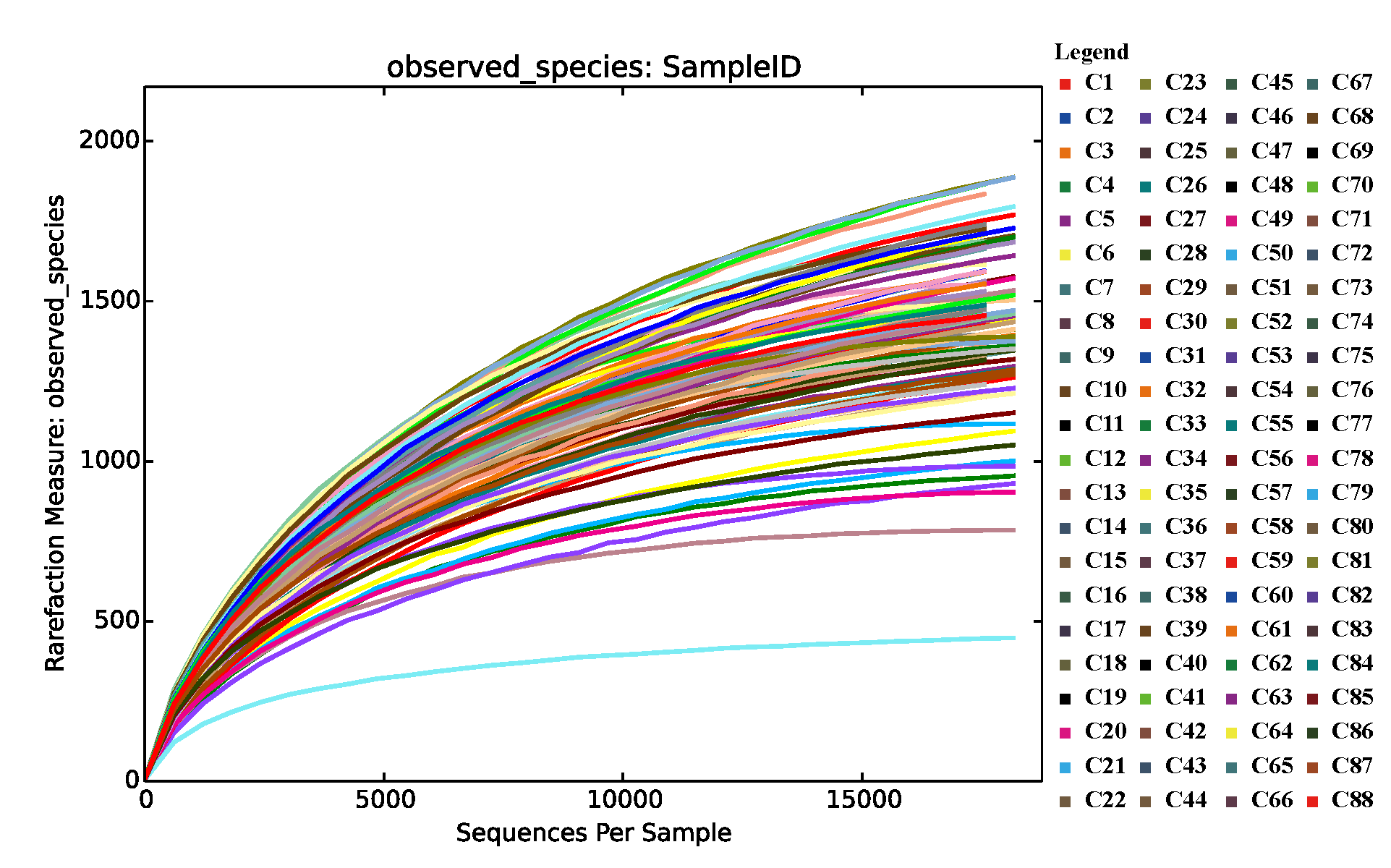
**

**Figure S2.**


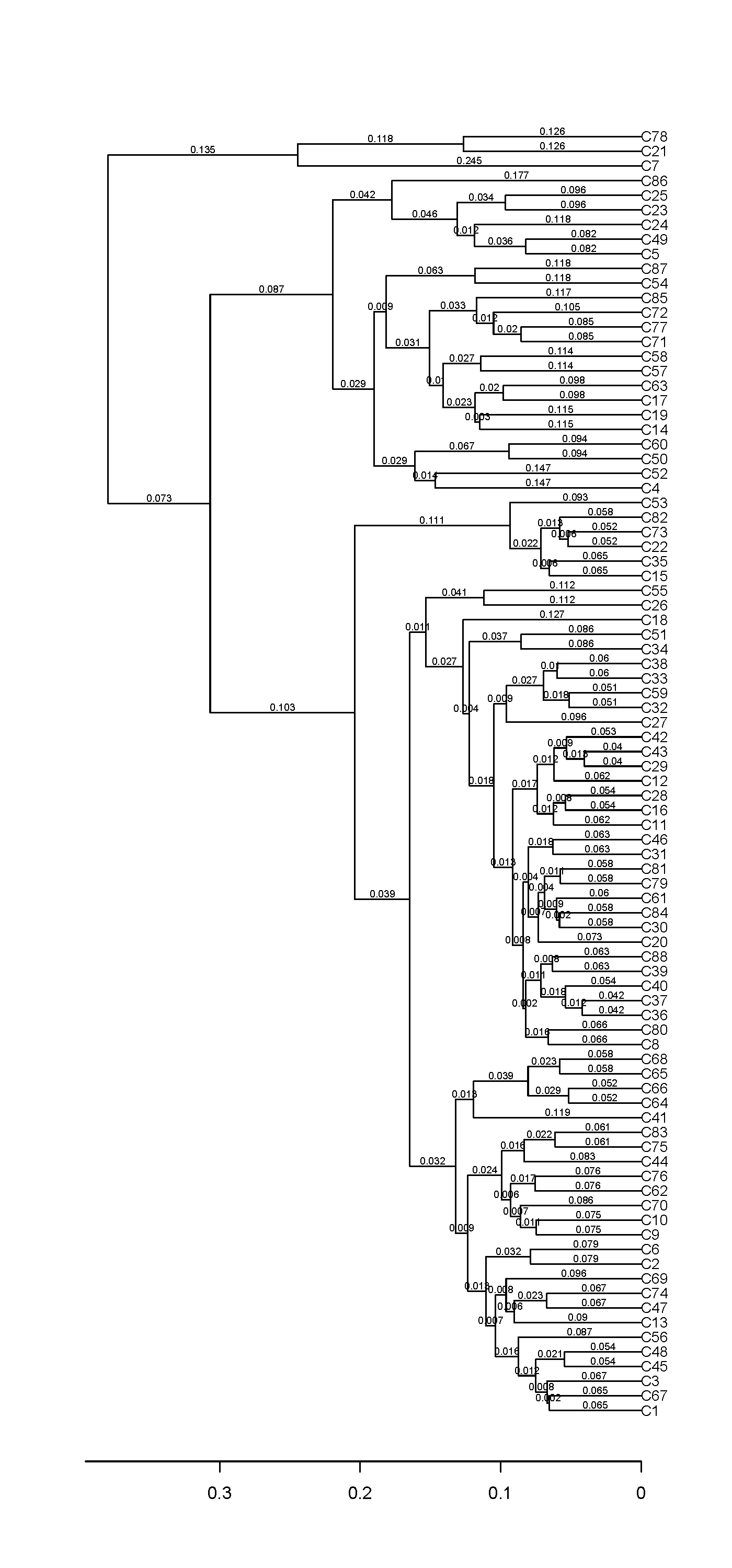

Supplement: Supplementary file 1 — Additional file 1: Figure S1. Rarefaction curves of the number of operational taxonomic units (OTUs) at 97% similarity boxplot for each of 88 samples. Figure S2. UPGMA result based on the unweighted Unifrac metric. The hierarchical clustering structure helps to determine the similarity of the bacterial communities between different samples. [file 12866_2021_2252_MOESM1_ESM.docx]
